# Analysis of oxybutynin treatment for hyperhidrosis in patients aged over 40 years

**DOI:** 10.1590/S1679-45082014AO2841

**Published:** 2014

**Authors:** Nelson Wolosker, Mariana Krutman, Marcelo Passos Teivelis, Rafael Pessanha de Paula, Paulo Kauffman, Jose Ribas Milanez de Campos, Pedro Puech-Leão

**Affiliations:** 1 Hospital Israelita Albert Einstein, São Paulo, SP, Brazil; 2 Hospital das Clínicas, Faculdade de Medicina, Universidade de São Paulo, São Paulo, SP, Brazil

**Keywords:** Hyperhidrosis/therapy, Botulinum toxins/therapeutic use, Quality of Life, Aged, Pharmacology

## Abstract

**Objective:**

Our aim was to analyze the effectiveness of oxybutynin for hyperhidrosis treatment in patients over 40 years.

**Methods:**

Eighty-seven patients aged over 40 years were divided into two groups. One group consisted of 48 (55.2%) patients aged between 40 and 49 years, and another was composed of 39 (44.8%) patients aged over 50 years (50 to 74 years). A comparative analysis of Quality of Life and level of hyperhidrosis between the groups was carried out 6 weeks after a protocol treatment with oxybutynin. A validated clinical questionnaire was used for evaluation.

**Results:**

In the younger age group, 75% of patients referred a “partial” or “great” improvement in level of hyperhidrosis after treatment. This number was particularly impressive in patients over 50 years, in which 87.2% of the cases demonstrated similar levels of improvement. Over 77% of patients in both groups demonstrated improvement in Quality of Life. Excellent outcomes were observed in older patients, in which 87.1% of patients presented “slightly better” (41%) or “much better” (46.1%) improvement.

**Conclusion:**

Patients aged over 40 years with hyperhidrosis presented excellent results after oxybutynin treatment. These outcomes were particularly impressive in the age group over 50 years, in which most patients had significant improvement in Quality of Life and in level of hyperhidrosis.

## RESUMO

**Objetivo:**

Analisar a efetividade da oxibutinina para tratamento da hiperidrose em pacientes com mais de 40 anos.

**Métodos:**

Oitenta e sete pacientes com idade superior a 40 anos foram divididos em dois grupos: o primeiro com 48 pacientes (55,2%), com idades entre 40 e 49 anos. O segundo com 39 pacientes (44,8%), com mais de 50 anos (intervalo: 50 a 74 anos). Uma análise comparativa da Qualidade de Vida e do nível de hiperidrose entre os grupos foi realizada 6 semanas após o início do tratamento com oxibutinina. Para isso, foi utilizado um questionário validado para Qualidade de Vida.

**Resultados:**

No grupo mais jovem, 75% dos pacientes referiram melhora “parcial” ou “ótima” no nível de hiperidrose após o tratamento. Esse número foi particularmente impressionante em pacientes acima de 50 anos, sendo que 87,2% apresentaram níveis similares de melhora. Mais de 77% dos pacientes, em ambos os grupos, demonstraram melhora na Qualidade de Vida. Desfechos excelentes foram observados em pacientes mais idosos, dentre os quais 87,1% dos pacientes apresentaram melhora “pouco melhor” (41%) ou “muito melhor” (46,1%).

**Conclusão:**

Os pacientes acima de 40 anos com hiperidrose tiveram excelentes resultados com o tratamento com oxibutinina. Esses desfechos foram particularmente importantes nos pacientes com mais de 50 anos, nos quais a maioria apresentou melhora da Qualidade de Vida e da hiperidrose.

## INTRODUCTION

Primary hyperhidrosis is a condition known for excessive sweating, beyond the body's physiologic needs^(1)^ that impacts in Quality of Life (QOL).^(2)^ A greater knowledge of the disease associated with upcoming therapeutic possibilities increased patient demand for treatment. The main areas affected are axilla, palm of hands, sole of feet and face.

Primary hyperhidrosis may manifest during childhood^(3)^ and persists throughout adult life. Epidemiologic studies and clinical practice demonstrate a greater prevalence among young, working age individuals, who are most commonly affected, and often seek healthcare professionals.

Non-invasive treatment possibilities include topical agents, application of botulinum toxin and iontophoresis, all presenting restricted effectiveness. Video-assisted thoracoscopy sympathectomy (VATS) is currently considered the gold standard treatment for hyperhidrosis; however, surgical risks and possibility of compensatory hyperhidrosis are the main limiting factors.^(4)^ Elderly patients are especially concerned with perioperative risk, and usually prefer not to be treated with invasive methods.

It was demonstrated that patients with primary hyperhidrosis have a higher expression of acetylcholine and alpha-7 nicotinic receptors in sympathetic ganglia.^(5)^ Oxybutynin is an antimuscarinic drug that was first associated to hyperhidrosis in 1988.^(6)^ In the second half of the last decade, specific treatment with this drug began to be reported.^(7,8)^ Its use was studied for specificsite hyperhidrosis: axillary,^(9)^ facial,^(10)^ palmar^(11)^ and plantar,^(12)^ all with good results. The first randomized placebo-controlled trial was reported in 2012,^(13)^ for analysis of the initial treatment of palmar and axillary hyperhidrosis, with improvement in over 70% of patients when compared to placebo.

This medication has been increasingly used as an initial or alternative therapy, especially in older patients who are not candidates for surgery. Studies analyzing benefit of this medication for patients over 40 years have not been carried out.

## OBJECTIVE

To analyze effectiveness of treatment (in levels of sweating) and improvements in Quality of Life of patients aged over 40 years, treated with low doses of oxybutynin.

## METHODS

This was a retrospective analysis based on medical charts of 87 patients aged over 40 years, carried out from January 2007 to December 2011, in two tertiarycare hospitals in São Paulo (*Hospital das Clínicas* and *Hospital Israelita Albert Einstein*). All patients are routinely followed in an outpatient clinic and treated in accordance to a protocol that will be further discussed. This study was approved by the Ethics Committee (CAAE 01582112.6.1001.0071).

The patients were divided into two groups: the first group (Group 40-49) consisted of 48 patients with age range 40-49 years, and the second group (Group ≥50) was composed of 39 patients aged over 50 years, range 50-74 years. Patients were not excluded based on pre-treatment regular medications.

A protocol treatment was applied to all patients consisting of an initial administration of 2.5mg/day of oxybutynin, during the first week. From the 8th to 21st day, the dose was doubled to 2.5mg twice a day, and from the 22nd day to the end of the 6th week, 5mg was administered twice daily. Our experience has showed that a progressively increased administration reduces the impact of anticholinergic side effects and increases tolerance to medication.

Clinical and QOL improvements were analyzed in two different moments during the study. The first evaluation was performed pre-treatment and the second, after 6 week medical therapy. Clinical improvement was classified using a scale ranging from zero to 10, where zero represented no improvement, and 10 accounted for absence of hyperhidrosis. The clinical improvement was ranked according to the following categories: null (from zero to 4), partial (5 to 7) or great (8 to 10).

QOL analysis was based on a validated clinical protocol questionnaire^(14–16)^ applied at each visit. Unspecific methods for QOL assessment do not permit a precise analysis of patients with hyperhidrosis, since relevant information for this disorder is not commonly addressed. The QOL questionnaire used in this study was validated and used in several publications (Appendix 1). Questions focus on influence of hyperhidrosis in different daily life situations, involving social, emotional and professional activities. Scores were given according to the patient's subjective perception of hyperhidrosis improvement, without any form of examiner interference by opinions.

QOL before treatment was classified into five different satisfaction categories, calculated as the added total score from the protocol (ranging from 20 to 100). The scoring system was designed in such a way that greater scores reflect more significant impact, representing poorer QOL. When the total score was greater or equal to 84 QOL was considered “very poor”. For scores ranging from 68 to 83, QOL was classified as “poor”. Scores that ranged from 52 to 67 were considered “good”. Scores from 36 to 51 indicated “very good” results, and scores from 20 to 35 were considered “excellent”.

Similarly, improvement in QOL after treatment was also classified into five different levels. When the total score was greater or equal to 84 the QOL was considered worse. When the scores ranged from 68 to 83, QOL was considered slightly worse. Scores from 52 to 67 were considered unaltered. Scores from 36 to 51 indicated a slightly better improvement, and from 20 to 35 were considered much better.

In both studied groups, the final outcomes analyzed were clinical improvement of hyperhidrosis and progress in QOL after treatment.

The χ^2^ test was performed to verify the association between categorical variables in contingency tables. The significance level considered for all statistical tests was 0.05.

## RESULTS

Age, site of hyperhidrosis, body mass index (BMI) distribution and gender of the studied population are demonstrated on table 1.

**Table 1:** Age, site of hyperhidrosis, body mass index and gender distribution in the study population

Variable	Category measures	40-49 years	≥50 years	p value*
Age	n	48	39	
	Range (min-max)	40-49	50-74	
	Mean (±SD)	43.2 (±2.59)	57.3 (±6.09)	
	Median	43	57	
Site of	Axillary	29 (60.4)	10 (25.6)	<0.0001
hyperhidrosis,	Palmar	9 (18.7)	6 (15.4)	
n (%)	Facial	8 (16.6)	22 (56.4)	
	Plantar	2 (4.3)	1 (2.6)	
BMI, n (%)	<25	22 (73.3)	14 (35.9)	0.11
	≥25	26 (26.7)	25 (64.1)	
Gender, n (%)	Female	33 (75.4)	25 (64.1)	0.16
	Male	15 (24.6)	14 (35.9)	

* χ^2^ test.

Min: minimum; max: maximum; SD: standard deviation; BMI: body mass index.

Female gender prevailed in both age groups. The predominant site of hyperhidrosis in younger patients (40 to 50 years) was axilla (60.4%), whereas in patients over 50 years, it was the face (56.4%). BMI values were higher (≥25kg/m^2^) in patients over 50 years.

All patients enrolled in the study classified their QOL before treatment as “poor” or “very poor”, as demonstrated on table 2. There was no statistically significant difference between the groups.

**Table 2:** Quality of Life assessment before treatment

QOL before treatment	Score	40-49 years n (%)	≥50 years n (%)	p value*
Very poor	84-100	27 (56.2)	24 (61.5)	
Poor	68-83	21 (43.8)	15 (38.5)	
Good	52-67	–	–	0.42
Very good	36-51	–	–	
Excellent	20-35	–	–	
Total		48	39	

* χ^2^ test.

QOL: Quality of Life.

Clinical improvement in hyperhidrosis, according to a subjective evaluation based on the patients' perception of their symptoms is shown on table 3. In the younger age group, 75% of patients referred a “partial” or “great” improvement in level of hyperhidrosis after treatment. This number was particularly impressive in patients over 50 years, in which 87.2% of the cases demonstrated similar levels of progress.

**Table 3:** Clinical improvement in hyperhidrosis after treatment

Treatment result	Score	40-49 years n (%)	≥50 years n (%)	p value*
No improvement	0-4	12 (25.0)	5 (12.8)	
Partial improvement	5-7	11 (22.9)	12 (30.7)	0.33
Great improvement	8-10	25 (52.1)	22 (56.5)	
Total		48	39	

* χ^2^ test comparing “no improvement” with “partial improvement and “great improvement”.

The improvement in QOL after 6 weeks of treatment with oxybutynin is displayed on table 4. Over 77% of patients in both groups demonstrated improvement in QOL (“much better” or “slightly better”). Again, especially good outcomes were observed in older age patients, in which 87.1% of them presented “slight” (41.0%) or “much better” (46.1%) improvement.

**Table 4:** Quality of Life after treatment

Treatment result	Score	40-49 years n (%)	≥50 years n (%)	p value*
Much better	20-35	20 (41.7)	18 (46.1)	
Slightly better	36-51	16 (33.3)	16 (41.0)	
Unaltered	52-67	12 (25.0)	5 (12.9)	0.38
Slightly worse	68-83	-	-	
Much worse	≥84	-	-	
Total		48	39	

* χ^2^ test comparing “unaltered” with “much better” and “slightly better”.

The only side effect associated with oxybutynin was dry mouth, observed in 72 patients (82.7%); however none required discontinuation of treatment. The distribution of this adverse event according to age is presented on table 5. Both groups presented similar incidence rates and intensity of dry mouth.

**Table 5:** Intensity of side-effects (dry mouth) according to age group

Treatment result	40-49 years n (%)	≥50 years n (%)	p value*
Absent	8 (16.6)	7 (18.0)	
Mild	9 (18.8)	11 (28.2)	0.36
Moderate	13 (27.1)	13 (33.3)	

Severe	18 (37.5)	8 (20.5)	
Total	48	39	

* χ^2^ test comparing “absent” and “light” dry mouth with “moderate” and “severe”.

## DISCUSSION

Primary hyperhidrosis is a disorder that essentially affects young individuals, who desperately seek medical assistance willing to undergo any treatment available to improve their QOL. Prevalence of this disorder decreases with ageing, especially after 40 years.^(17)^ This could result from a reduced treatment demand from this population who usually improved severity of symptoms with age, or simply adapted to their condition. However, approximately 3%^(10)^ of general population suffers from excessive sweating and may be unaware that effective treatment alternatives are available.

Particular epidemiologic characteristics in hyperhidrosis patients over 40 years can be outlined with our research results. It is well known that in general population, hyperhidrosis prevalence rates are higher in females,^(18)^ especially as a consequence of the esthetic and social concerns involved, which are particularly disturbing for women. Similarly, both our study groups disclosed a predominance of the female sex, but the proportion of male individuals increases with age. This may result from a reduced esthetic interest noted in older women.

Distribution of hyperhidrosis also differs in older individuals, and the face is the most frequent site.^(2,19)^ This is consistent with our results that presented a 56.4% incidence of facial hyperhidrosis in patients over 50 years. The good results with oxybutynin treatment in patients with craniofacial hyperhidrosis demonstrated in previous studies^(19)^ corroborate with overall excellent outcomes obtained in our sample population. Amelioration of other sites of hyperhidrosis was similar between genders in our sample population, as has been demonstrated to be on unselected population treated with oxybutynin.^(20)^

Gallagher et al.^(21)^ have demonstrated that body fat percentage increases with age for both men and women, and these changes occur as a result of alterations in body composition related to the process of ageing. This explains the higher proportion of patients with BMI greater than 25mg/kg^2^ observed in the age group over 50 years. In the general population, improvement with oxybutynin is comparable between normal and overweight/obese patients.^(22)^

The excellent results obtained with oxybutynin treatment in patients over 40 years were comparable to surgical outcomes. VATS is currently considered the best treatment modality and is effective for hyperhidrosis in approximately 95%^(23,24)^ of cases, however with an up to 30%^(4)^ risk of compensatory hyperhidrosis. Considering that 87.2% of the individuals over 50 years presented “slight” or “great” improvement in hyperhidrosis, and 87.1% significantly improved QOL, we could argue that medical therapy can be a good alternative for this specific age group.

Oxybutynin is an anticholinergic drug widely used for treating urologic conditions, such as overactive bladder. The only formal contraindication is closed angle glaucoma. Apart from this concern, it is a safe medication but with limited tolerability due to antimuscarinic side effects, especially noted with the administration of doses over 15mg/day.^(25)^ The maximum dose of 10mg/ day associated with the slow and progressive increase in dosage used in our protocol, lowers the incidence of side effects, maintaining effectiveness and improving compliance to treatment. Dry mouth was the only side effect observed in our series, and no patient required discontinuation of treatment due to adverse events.

Regarding our patient's age group, it should be noted that elderly individuals usually take multiple drugs simultaneously,^(26)^ and that their metabolism, pharmacokinetics and pharmacodynamics may be altered.^(27)^ While we believe the complaint of hyperhidrosis was primary on all cases, some patients may have failed to report concomitant use of drugs, such as antidepressants that may be associated with both hyperhidrosis^(28)^ and hypohidrosis.^(29)^ Nevertheless, we believe that the trend of overall improvement in most patients is attributable to the anticholinergic drug administered: it is, in our opinion, highly unlikely that a significant number of patients would have started other drugs during the 6 week period of our evaluation, and their improvement in hyperhidrosis could be erroneously attributed to oxybutynin.

## CONCLUSION

Patients aged over 40 years with hyperhidrosis present excellent results with oxybutynin treatment. These outcomes are particularly impressive in the age group over 50 years, in which over 87% of patients had significant improvement in Quality of Life and in level of hyperhidrosis.
